# Supplementary material for: The Human Pathogen Mycobacterium tuberculosis and the Fish Pathogen Mycobacterium marinum Trigger a Core Set of Late Innate Immune Response Genes in Zebrafish Larvae
Source: Biology (Basel). 2024 Sep 3;13(9):688. doi: 10.3390/biology13090688 (PMC11429319; doi:10.3390/biology13090688)
Supplement: Supplementary file 1 [file biology-13-00688-s001.zip › Supplemental figures_2024 September_final.pdf]

Supplemental Figures

The Human Pathogen *Mycobacterium tuberculosis* and the Fish Pathogen *Mycobacterium marinum* Trigger a Core Set of Late Innate Immune Response Genes in Zebrafish Larvae

Ron P. Dirks [1,*,†], Anita Ordas [1], Susanne Jong-Raadsen [1], Sebastiaan A. Brittijn [1], Mariëlle C. Haks [2], Christiaan V. Henkel [1], Katarina Oravcova [3], Peter I. Racz [1], Nynke Tuinhof-Koelma [1], Malgorzata I. Korzeniowska nee Wiweger [1], Stephen H. Gillespie [4], Annemarie H. Meijer [5], Tom H. M. Ottenhoff [2], Hans J. Jansen [1,†] and Herman P. Spaink [5,*]

Figure S1. (A) Statistical analysis of the survival experiment of Figure 2. The following outcome was obtained: uninjected and PVP controls are not different; PVP control and wt H37rv are different and P=0.0047; PVP control and Mtb-DsRED are different and P< 0.0001; wt H37rv and Mtb-DsRED are also different P <0.0001; uninjected and wt H37rv are not different; uninjected and Mtb-DsRED are different p<0.0001. Raw Data is presented in Supplemental table 4. (B) Survival experiment after robotic injection with *M. marinum* in zebrafish larvae using the same set up as for *M. tuberculosis* except that the temperature was set at 28 degrees Celsius. In total 100 embryos were injected with 50 CFU *M. marinum* strain M according to published methods [20]. At the start of the experiment the larvae were supplied with 200 uM rifampicin and 2 mM Isoniazid in DMSO. As a control the solvent DMSO was added to the medium at a concentration of 0.4%. The mortality in the control due to the infection by *M.marinum* is conform earlier published results from our group [30]. The rescue by 200 uM rifampicin and 2 mM Isoniazid shows that there is no mortality due to the injection procedure or quality of the larvae.

A

B

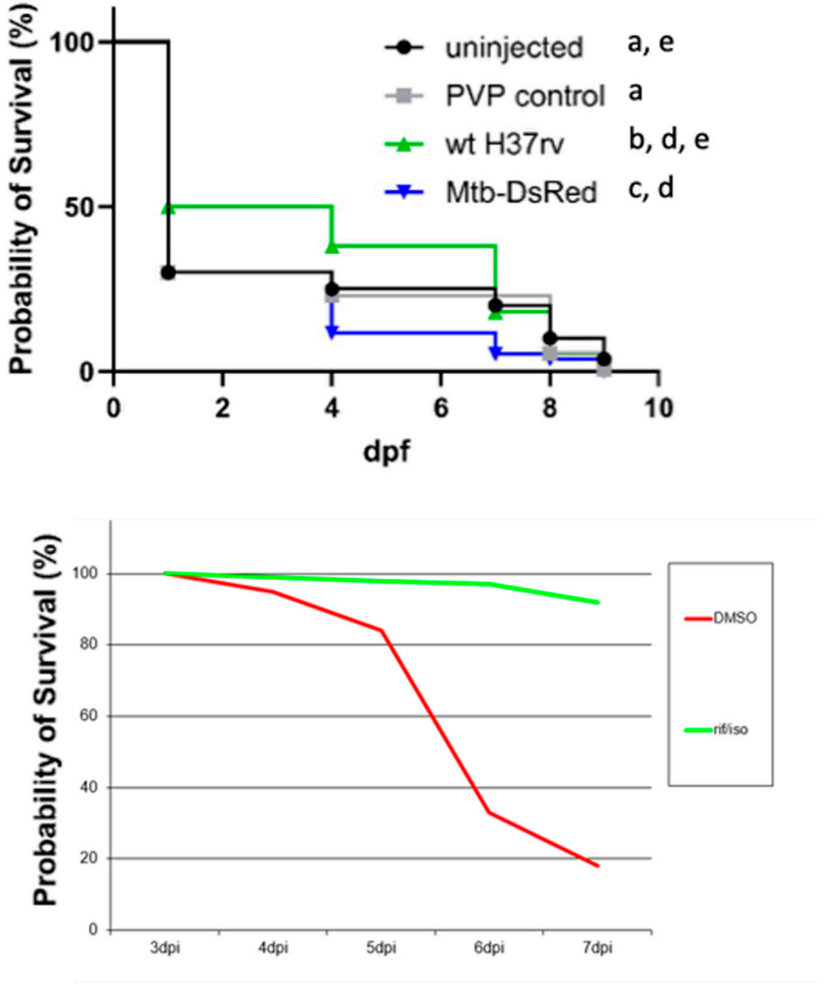

Probability of Survival (%)
100
50
0
0
2
4
6
8
10
dpf
uninjected
a, e
PVP control
a
wt H37rv
b, d, e
Mtb-DsRed
c, d
Probability of Survival (%)
100
80
60
40
20
0
3dpi
4dpi
5dpi
6dpi
7dpi
DMSO
rif/iso

Figure S2. The data of Figure 4 shown on a Log scale.

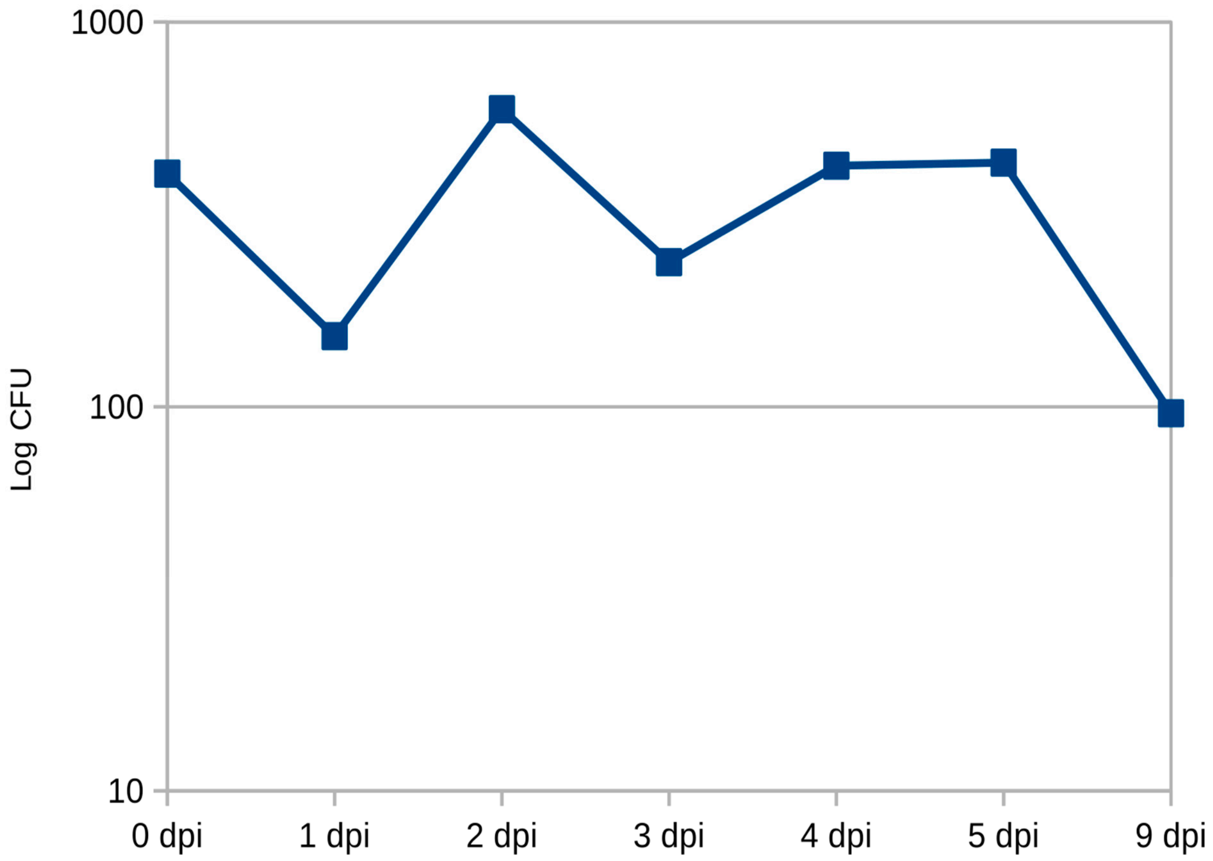

Figure S3. Statistical analysis of the data from Figure 5. For *Il1b* the combined data of 5 and 6 days post infection appeared significant with a Mann Whitney test with p value of 0.025 (test statistics 4, critical value 5). For *mmp9* the combined data of 3 and 4 days and 5 and 6 days appeared significant with p values of 0.016 (test statistics 3, critical value 5) and 0.006 (test statistics 1, critical value 5), respectively.

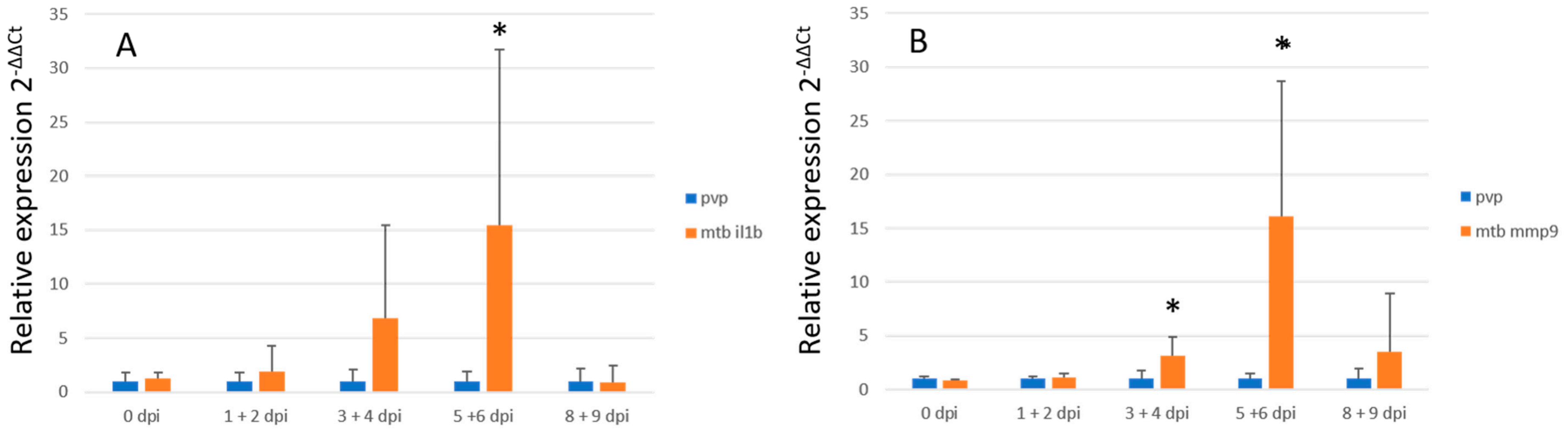

Figure S4. Infection-independent comparison of the transcriptome profiles of the *M.marinum* and *M.tuberculosis* experiments using Venn diagrams.

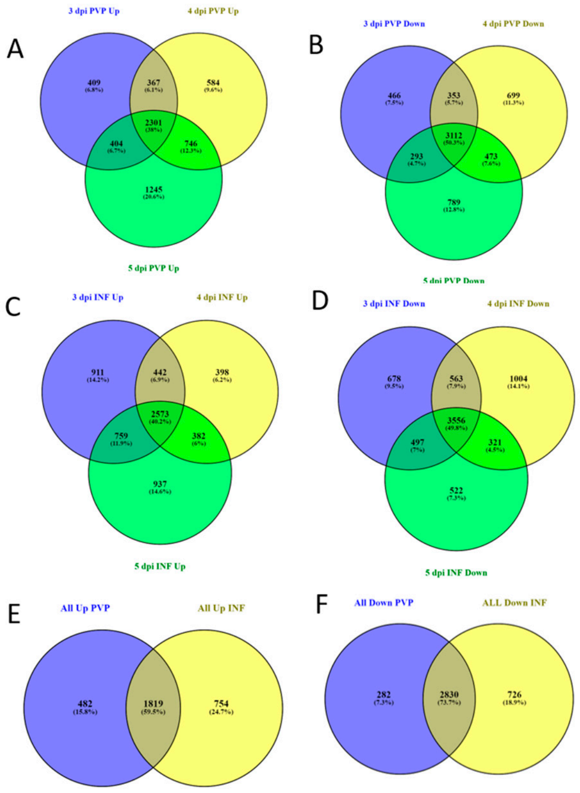

Figure S5. GO enrichment analysis of the 1819 common up-regulated infection-independent genes shown in Figure S4.

Sublist	Category	Term	RT	Genes	Count	%	P-Value	Benjamini
	KEGG_PATHWAY	Cytoskeleton in muscle cells	RT		51	3.7	2.9E-10	4.7E-8
	KEGG_PATHWAY	Motor proteins	RT		33	2.4	3.6E-5	2.9E-3
	KEGG_PATHWAY	ECM-receptor interaction	RT		15	1.1	3.3E-3	1.7E-1
	KEGG_PATHWAY	Regulation of actin cytoskeleton	RT		32	2.3	4.5E-3	1.7E-1
	KEGG_PATHWAY	Adrenergic signaling in cardiomyocytes	RT		26	1.9	6.1E-3	1.7E-1
	KEGG_PATHWAY	Focal adhesion	RT		28	2.0	6.3E-3	1.7E-1
	KEGG_PATHWAY	Vascular smooth muscle contraction	RT		20	1.4	8.0E-3	1.8E-1
	KEGG_PATHWAY	Lysosome	RT		20	1.4	1.1E-2	2.2E-1
	KEGG_PATHWAY	Phenylalanine metabolism	RT		5	0.4	1.2E-2	2.2E-1
	KEGG_PATHWAY	Arginine and proline metabolism	RT		10	0.7	1.6E-2	2.6E-1
	KEGG_PATHWAY	Phagosome	RT		19	1.4	2.0E-2	2.9E-1
	KEGG_PATHWAY	Cardiac muscle contraction	RT		16	1.2	2.3E-2	3.1E-1
	KEGG_PATHWAY	Metabolic pathways	RT		128	9.3	2.8E-2	3.2E-1
	KEGG_PATHWAY	Ferroptosis	RT		9	0.7	2.8E-2	3.2E-1
	KEGG_PATHWAY	Calcium signaling pathway	RT		33	2.4	3.5E-2	3.5E-1
	KEGG_PATHWAY	Apelin signaling pathway	RT		19	1.4	3.5E-2	3.5E-1
	KEGG_PATHWAY	alpha-Linolenic acid metabolism	RT		5	0.4	3.7E-2	3.5E-1
	KEGG_PATHWAY	Porphyrin metabolism	RT		10	0.7	3.9E-2	3.5E-1
	KEGG_PATHWAY	Ether lipid metabolism	RT		8	0.6	4.5E-2	3.7E-1
	KEGG_PATHWAY	Galactose metabolism	RT		6	0.4	4.7E-2	3.7E-1
	KEGG_PATHWAY	Ascorbate and aldarate metabolism	RT		8	0.6	4.9E-2	3.7E-1
	KEGG_PATHWAY	Phenylalanine, tyrosine and tryptophan biosynthesis	RT		3	0.2	6.0E-2	4.3E-1
	KEGG_PATHWAY	Starch and sucrose metabolism	RT		6	0.4	7.0E-2	4.7E-1
	KEGG_PATHWAY	Arachidonic acid metabolism	RT		8	0.6	7.1E-2	4.7E-1
	KEGG_PATHWAY	Sphingolipid metabolism	RT		9	0.7	7.3E-2	4.7E-1
	KEGG_PATHWAY	Tyrosine metabolism	RT		6	0.4	7.7E-2	4.7E-1
	KEGG_PATHWAY	Linoleic acid metabolism	RT		5	0.4	8.8E-2	5.2E-1
